# Brain targeting of zolmitriptan via transdermal terpesomes: statistical optimization and in vivo biodistribution study by ^99m^Tc radiolabeling technique

**DOI:** 10.1007/s13346-023-01373-0

**Published:** 2023-06-05

**Authors:** Mai Ahmed Tawfik, Mai M. Eltaweel, Ahmed M. Fatouh, Hesham A. Shamsel-Din, Ahmed B. Ibrahim

**Affiliations:** 1 Department of Pharmaceutics and Industrial Pharmacy, Faculty of Pharmacy, Cairo University, Cairo, Egypt; 2 Labeled Compounds Department, Hot Labs Center, Egyptian Atomic Energy Authority, Cairo, 13759 Egypt

**Keywords:** Zolmitriptan, Transdermal, Terpesomes, Radiolabeling, Brain Targeting

## Abstract

Zolmitriptan (ZT) is a potent second generation triptan, commonly administered to alleviate migraine attacks. ZT suffers various limitations; massive hepatic first pass metabolism, P-gp efflux transporters susceptibility, and limited (≈40%) oral bioavailability. Transdermal route of administration could be explored to enhance its bioavailability. A 2^3^.3^1^ full factorial design was constructed to developed twenty-four ZT loaded terpesomes via thin film hydration technique. The influence of drug: phosphatidylcholine ratio, terpene type, terpene concentration and sodium deoxycholate concentration on the characterization of the developed ZT-loaded terpesomes was assessed. Particle size (PS), zeta potential (ZP), ZT entrapment efficiency (EE%), drug loading (DL%) and drug released percentages after 6 h (Q_6h_) were the selected dependent variables. Further morphological, crystallinity, and in-vivo histopathological studies were conducted for the optimum terpesomes (T6). ^99m^Tc-ZT and ^99m^Tc-ZT-T6 gel were radio-formulated for in-vivo biodistribution studies in mice following transdermal application of ^99m^Tc-ZT-T6 gel, relative to ^99m^Tc-ZT oral solution. T6 terpesomes [comprising ZT and phosphatidylcholine (1:15), cineole (1% w/v) and sodium deoxycholate (0.1% w/v)] were optimum with respect to spherical PS (290.2 nm), ZP (-48.9 mV), EE% (83%), DL% (3.9%) and Q_6h_ (92.2%) with desirability value of 0.85. The safety of the developed T6 terpesomes was verified by the in-vivo histopathological studies. ^99m^Tc-ZT-T6 gel showed maximum brain concentration (5 ± 0.1%ID/ g) with highest brain to blood ratio of 1.92 ± 0.1 at 4 h post transdermal application. Significant improvement of ZT brain relative bioavailability (529%) and high brain targeting efficiency (315%) were revealed with ^99m^Tc-ZT-T6 gel, which confirmed successful ZT delivery to the brain. Terpesomes could be safe, successful systems capable of improving ZT bioavailability with high brain targeting efficiency.

**Graphical Abstract:**

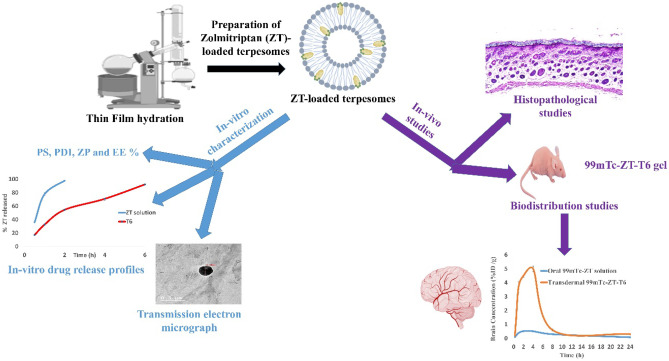

## Introduction

Migraine is a neurological disorder resulting in a special type of headache that affects one side of the head with pulsating pain. It may be associated with aura which is visual disturbance causing patient confusion [1]. According to the World Health Organization (WHO), migraine is the most prevalent long-term neurological condition causing patient disability [2]. Migraine is usually coupled with atypical serotonergic activity, and it is thought to originate from secretion of vasodilator peptides that induce dilation of the meningeal arteries and perivascular inflammation [3]. Zolmitriptan (ZT) is a second generation triptan that constricts the dilated blood vessels in migraine via stimulating the serotonin 5-HT_1B/1D_ receptors [4] and thereby inhibiting the release of vasoactive peptides. ZT suffers various limitations that reduce its oral bioavailability to ≈ 40%. Firstly, it belongs to class III according to the biopharmaceutics classification system (BCS). ZT has limited gastrointestinal tract (GIT) permeability probably due to its efflux by P-gp transporters [5]. Secondly, it is subjected to hepatic first pass metabolism. Thirdly, reduced oral absorption of therapeutics is often revealed when administrated by migraine patients who often have GIT disturbance, nausea and vomiting [6]. This limited oral bioavailability urged utilizing alternative routes of administration. Among the routes that have been investigated to achieve higher systemic bioavailability of ZT; the intranasal [7], transdermal [8], as well as buccal routes [9].

The transdermal route is characterized by numerous points of strength that can enhance ZT bioavailability; i) it conveys drug directly into the systemic circulation avoiding the portal circulation and consequently first pass metabolism, ii) it avoids the GIT which diminishes the negative effect of the migraine associated GIT disturbance on ZT bioavailability, and iii) it provides consistent drug levels in blood via controlled delivery, that decreases the dosing frequency as well as associated side effects [10]. Stratum cornuem (SC), the upper layer of skin, represents the main barrier for the transdermal absorption of drugs [11]. Therefore, utilizing penetration enhancer that facilitates drug permeation through the SC is considered crucial to accomplish successful transdermal absorption.

Terpenes are natural compounds characterized by skin penetration enhancing effect. They have been utilized in the development of a novel ultra-deformable vesicular system called “Terpesomes”. Terpesomes can be described as nano-vesicular systems consisting of phospholipid(s), ethanol in addition to one or more of terpenes [12, 13]. In terpesomes, the phospholipid constitutes the vesicular bilayer matrix while the bilayer-imbedded ethanol and terpenes impart fluidity to vesicular wall. This ultra-deformable feature is thought to enable terpesomes to compress themselves through the SC narrow pores and traverse it more abundantly. Moreover, terpenes along with ethanol are believed to disrupt the SC intercellular lipids facilitating the intercellular transport of drugs through the SC. In addition, terpenes and ethanol influence on SC structure can improve drug partitioning into skin allowing more drug to partition into skin which creates increased driving force for skin penetration into the systemic circulation [14, 15].

Radiolabeling is an effective technique in tracing and detecting drugs in the in-vivo biodistribution studies. The radiolabeling can be accomplished via; i) linking the formulation to a radio-active tracer [16], or ii) radio-labeling the drug molecule then incorporating it into the formulation [17–19].

Previous studies explored the transdermal route of administration for enhancing the bioavailability of ZT via bypassing portal and liver metabolism through the development of various systems as nanostrucutured lipid carriers [20], niosomal emulgel [8], adhesive patch [21]. On the other hand, successful skin permeation of various drugs was achieved via terpesomes [22–25]. Transdermal terpesomal ZT was not investigated before. We can summarize our research goal in exploring the potential of terpesomes for safe, effective and enhanced transdermal delivery of ZT. A 2^3^.3^1^ full factorial design was constructed to study the influences of the formulation variables on the terpesomes’ particle size, zeta potential, entrapment efficiency, drug loading and drug release. Histopathological study was adopted to test the safety of the terpesomes for transdermal application. Subsequently, ^99m^Tc was conjugated to the optimized terpesomal system. The biodistribution and pharmacokinetics of ZT in blood, brain and liver after transdermal application of the developed ^99m^Tc-optimized ZT terpesomal system were investigated and compared to an oral ^99m^Tc-ZT solution.

## Materials and methods

### Materials

Zolmitriptan (ZT) was donated by Global Nabi Pharmaceuticals (Giza, Egypt). L-α phosphatidylcholine from soybean oil (PC) was purchased from Acros Organics (Geel, Belgium). Terpenes (fenchone, cineole, and limonene), sodium deoxycholate (SDC), and sodium dithionite were attained from Sigma-Aldrich® (St. Louis, MO). Hydroxypropyl methylcellulose (HPMC; K4M) was bought from Dow Chemical Company (Midland, MI). Disodium hydrogen phosphate, sodium chloride, potassium dihydrogen phosphate, ethanol, methanol, and chloroform were purchased from El-Nasr Pharmaceutical Chemicals Co. (Cairo, Egypt). Technetium-99 m (^99m^Tc) elution was developed from ^99^Mo / ^99m^Tc generator as (^99m^TcO_4_^−^), Radioisotope Production Facility (RPF), Egyptian Atomic Energy Authority (EAEA) (Cairo, Egypt). Spectra Por^©^ dialysis membrane tubing (Mol. weight cut off; 14 g/mol) was acquired from Spectrum Laboratories Inc. (California, USA). All other chemicals (analytical grade) were utilized as received.

### Preparation of ZT-loaded terpesomes

Thin film hydration technique was adopted for preparing twenty four ZT-loaded terpesomes using four different variables; (i) drug: PC ratio (1:15 or 1:7.5), (ii) terpene type (fenchone, cineole, or limonene), (iii) terpene concentration (1% or 2%, w/v) and SDC concentration (0% or 0.1%, w/v) [22, 23]. Firstly, ZT (20 mg), PC, terpene and SDC (if present) were solubilized in an organic solvent mixture (chloroform: methanol; 2:1) in a flask via sonication (Crest ultrasonics corp., Trenton, USA). The organic mixture was removed and a clear thin film of terpesomes was obtained by maintaining reduced pressure at 120 rpm and 60 °C for 30 min, using a rotatory evaporator (Heidolph VV 2000, Burladingen, Germany). Following, efficient film hydration was achieved by adding the hydrating medium (3% v/v ethanolic distilled water; 10 mL), rotating the flask at 120 rpm under normal pressure and room temperature for 30 min, in the presence of small glass beads [26]. The terpesomal dispersions (final ZT concentration; 2 mg/mL) were kept overnight at 4 °C, for equilibrium and maturation.

### Preparation of ^99m^Tc-ZT solution and ^99m^Tc-ZT-T6 gel system

#### Preparation of ^99m^Tc-ZT solution

^99m^Tc-ZT solution was prepared via the direct labelling approach, under reductive conditions using sodium dithionite (Na_2_S_2_O_4_) as a reducing agent. 400 µL ZT solution (equivalent to 0.5–3 mg ZT) was added to 100 µL freshly eluted ^99m^Tc (100 MBq) and 400 µL freshly Na_2_S_2_O_4_ aqueous solution (equivalent to 1–35 mg of Na_2_S_2_O_4_). Adjusting the medium pH over the range 3–9 was adopted, utilizing 0.1 M HCl and/or 0.1 M NaOH aqueous solutions. The reaction mixture was completed to 1 mL by distilled water, stirred using an electrical vortex, and finally held for preselected time intervals at room temperature, prior to assessing the radiochemical yield (RCY).

#### Preparation of ^99m^Tc-ZT-T6

Under reductive conditions employing sodium dithionite (Na_2_S_2_O_4_), the optimum ZT-loaded terpesomes (T6) were directly radiolabel with ^99m^Tc. Different volumes (0.1–2.5 mL) of the optimum ZT-loaded terpesomes were utilized with various concentrations (0.5–15 mg/mL) of Na_2_S_2_O_4_ and the pH of the reaction mixture was adjusted to different pH values (3–9). A 0.1 mL aliquot of freshly eluted ^99^Mo/^99m^Tc generator technetium Tc-99 m pertechnetate (^99m^TcO_4_; 100 MBq) was added to the reaction mixture, which was then completed to 5 mL by distilled water. This procedure was conducted via shaking the reaction mixture for varying durations of reaction time at room temperature for 5–60 min.

For maximizing the radiochemical yield, the influence of varying different reaction conditions (Na_2_S_2_O_4_ concentration, ZT/ ZT-loaded terpesomes (ZT-T6) concentration, reaction medium pH, and reaction duration time) on the effectiveness of the radiolabeling technique was examined [27]. Experiments were carried out three times to examine the influence of each factor. One-way ANOVA test was used to assess the significance of difference of the results, at *p* < 0.05. Radiochemical yield (RCY) was determined utilizing thin layer chromatography (TLC). Acetone was employed as the mobile solvent for the determination of the percentage of the free ^99m^TcO_4_ as radio-impurities, whereas saline was used for the determination of the reduced hydrolyzed ^99m^TcO_2_ as radio-impurities. The radiochemical yield was calculated via the following equation:

1 $$\mathrm{The \;radiochemical \;yield }\left(\mathrm{RCY}\right)= 100\mathrm{\% }-\mathrm{ total \;radioimpurities\; \%}$$

#### Preparation of ^99m^Tc-ZT loaded terpesomal (^99m^Tc-ZT-T6) gel

The optimum ^99m^Tc-ZT loaded terpesomal gel was prepared by sprinkling 50 mg hydroxypropyl methylcellulose (HPMC) slowly on 10 mL of magnetically stirred ^99m^Tc-ZT-T6 (WiseStir, Daihan Scientific Company, Chhattisgarh, India), at 37 ± 1 °C. The developed ^99m^Tc-ZT-T6 gel was finally refrigerated, to form clear gel free from any obvious clumps or air bubbles.

### In-vitro characterization of ZT-loaded terpesomes

#### Determination of particle size (PS), polydispersity index (PDI) and zeta potential (ZP)

Photon correlation spectroscopy via Malvern Zetasizer Nano ZS (Worcestershine, UK) was adopted for measuring the mean PS of the ZT-loaded terpesomes at 25 ± 1 °C, after proper dilution (15 folds) with deionized distilled water [28]. PDI values were measured for the determination of the homogeneity of the particle size distribution. The ZP values of the negatively charged terpesomal vesicles were also assessed, via observing their electrophoretic mobility using the same equipment.

#### Determination of ZT entrapment efficiency (EE%)

The direct technique was conducted for the estimation of the EE%, DL% of ZT in the terpesomal dispersions [29]. Briefly, terpesomal dispersions (1 mL) were ultra-centrifuged, at 22,000 rpm (Sigma 3–30 KS, Sigma Laborzentrifugen GmbH, Germany) for 1 h at 4 °C. The residues, composed of the ZT-loaded terpesomes, were lysed and solubilized by sonication with ethanol (Crest ultrasonics corp., Trenton, USA). The entrapped ZT concentrations were assessed spectrophotometrically after appropriate dilution with ethanol (Shimadzu, model UV-1601 PC, Kyoto, Japan) at λ_max_ 285.6 nm [7]. In a parallel line, drug-free terpesomes were used as controls [22].

ZT EE% was calculated as follows;

2 $$\mathrm{EE\%}=\frac{\mathrm{Entrapped \;amount \;of \;ZT }\;(\mathrm{mg})}{\mathrm{Total \;amount \;of \;ZT }\;(\mathrm{mg})} \times 100$$

ZT DL% was calculated as follows;

3 $$\mathrm{DL\%}=\frac{\mathrm{Entrapped \;amount \;of \;ZT }\;(\mathrm{mg})}{\mathrm{Total \;weight \;of \;terpesomes }\;(\mathrm{mg})} \times 100$$

#### In-vitro ZT release studies

Semi-permeable membrane diffusion technique was applied for the determination of the release profiles of the developed ZT-loaded terpesomes. Aliquots of ZT-loaded terpesomes (containing 4 mg of ZT) were added to the pre-soaked cellulose dialysis bags, which were then securely closed and dipped in stoppered bottles containing the release media; 100 mL phosphate buffer saline (PBS; pH 7.4). The bottles were placed in a temperature controlled shaking water bath (Unimax, IKA, Germany), operated at 100 strokes per min at 37 ± 0.5 ºC [7]. Samples were withdrawn from the release media at different sampling points (0.5, 1, 2, 4, and 6 h) and analyzed spectrophotometrically at λ_max_ 285.6 nm. Simultaneously, fresh media (3 mL) were added to ensure sink condition and constant volume.

To ensure that the cellulose membrane would not hinder the release of ZT in the prementioned medium, in-vitro ZT release studies were also conducted for ZT aqueous solution [22]. The mean (± SD) ZT released percentages were calculated and plotted versus their respective time points. ZT released percentages after 6 h (Q_6h_) were determined for comparison.

#### Optimization of ZT-loaded terpesomes via 2^3^.3^1^ full factorial design

The influence of four independent formulation variables on the in-vitro characterization of the developed ZT-loaded terpesomes was studied via a 2^3^.3^1^ full factorial design, by utilizing Design-Expert^®^ software (Stat-Ease, Inc., Minneapolis, MN). Drug: PC ratio, terpene type, terpene concentration and SDC concentration were the independent variables. The responses were the PS, ZP, EE%, LD% and Q_6h_. Concerning the desirability constraints; (absolute value) ZP, EE%, LD% and Q_6h_ were set to be maximized, while PS was set to be minimized. Statistical analysis of data was adopted using one-way ANOVA at a *P* < 0.05, for the selection of the optimum ZT-loaded terpesomes. Post Hoc; least square difference (LSD) analysis was adopted, via SPSS^®^ software Ver. 19.0 (SPSS Inc., Chicago, USA), to estimate the significance of differences of the in-vitro characterization data of ZT-loaded terpesomes at *P* value < 0.05.

### Characterization of the optimum ZT-loaded terpesomes

#### Morphological examination via transmission electron microscopy (TEM)

A drop of the optimum ZT-loaded terpesomal dispersion was placed on a copper grid, and stained with phosphotungestic acid (1% w/v), dried and finally visualized at 80 kV using Joel JEM 1230 transmission electron microscope (Tokyo, Japan) [26, 30].

#### Crystallinity examination via differential scanning calorimetry (DSC) studies

The optimum ZT-loaded terpesomes were firstly prepared, frozen and finally lyophilized for 24 h (Novalyphe-NL 500 lyophilizer; Savant Instruments; NY, USA) under a reduced pressure of 7 × 10^−2^ mbar at –45 °C.

DSC analysis was adopted for the optimum lyophilized terpesomes, the physical mixture of all the solids components (ZT, PC and SDC), as well as each individual solid component via Shimadzu differential scanning calorimeter (DSC-60 Shimadzu, Kyoto, Japan). Samples were scanned in aluminum pans over a temperature range of 30–300 °C; at 10 °C/min heating rate, under nitrogen stream (20 mL/min), utilizing indium as a reference.

### In-vivo studies

#### In-vivo histopathological studies

The histopathological examination was adopted to test the biocompatibility and safety of the different components of terpesomes for transdermal application. Six Wistar mice (150–200 g each) were assigned randomly into 2 groups: first group acted as control, while the optimum terpesomes were tested on the second group (test group). The optimum ZT-loaded terpesomes were applied onto the skin of mice three times daily for a week [31]. Then ketamine was used to sacrifice the mice, and skin samples were excised for further examination as reported by Aziz et al. [10].

#### In-vivo biodistribution studies

The in-vivo biodistribution studies were conducted via a radiobiological technique by preparing oral ^99m^Tc-ZT solution and transdermal ^99m^Tc-ZT loaded terpesomal (^99m^Tc-ZT-T6) gel.

#### In-vivo biodistribution of oral ^99m^Tc-ZT solution and transdermal ^99m^Tc-ZT-T6 gel

The in-vivo biodistribution experimental protocol was reviewed and accepted by the Research Ethics Committee (PI-3022), Faculty of Pharmacy, Cairo University and Egyptian Atomic Energy Authority (EAEA 216/6–2022).

Forty-two Swiss albino male mice (20–27 g each) were divided randomly into two groups. Reference treatment (^99m^Tc-ZT solution) was orally administered to the first group, while test treatment (^99m^Tc-ZT-T6 gel) was applied transdermally to the second group. Each group was comprised of twenty-one mice (3 mice for each time point), placed in polypropylene cages, at 25 ^0^C with appropriate relative humidity (45–50%).

Mice of group A were administered specific volume of ^99m^Tc-ZT solution (equivalent to 20 µg/ g mice body weight of ZT; containing 5–10 MBq). On the other hand, appropriate volumes of ^99m^Tc-ZT-T6 gel (equivalent to 20 µg/ g mice body weight of ZT; containing 5–10 MBq) were applied to the muscle’s shaved skin of group B.

The in-vivo biodistribution study involved seven pre-designed time points up to 24 h (0.25, 0.5, 1, 2, 4, 8, and 24 h post oral administration or transdermal application). At each pre-designed time point, three mice from each group were weighed, anesthetized by chloroform and sacrificed. Blood samples were withdrawn via cardiac puncture [32]. The total blood weight was calculated, assuming that blood represents around 7% of the total body weight of mice [33, 34].

Brains as well as other organs were carefully separated, washed from any remaining tissue or blood by normal saline, and accurately weighed. ^99m^Tc-ZT concentrations in each organ and blood samples were counted by shielded gamma (γ) scintillation counter; NaI (TI) detector (Scalar ratemeter SR7; Nuclear Enterprises Ltd., Edinburgh, USA). At each pre-designed time point; the radiopharmaceutical uptake (detected as ^99m^Tc-ZT concentration) per gram of each organ or blood (%ID/ g) was calculated as a fraction of the orally administrated ^99m^Tc-ZT solution dose or the transdermally applied ^99m^Tc-ZT-T6 gel dose using the following equation [35].

4 $$\%\;\mathrm{ ID}/\mathrm{ g}=\frac{\mathrm{Sample\; radioactivity\; count\; X }\;100 }{\mathrm{Weight\; of\; sample }\left(\mathrm{g}\right)\mathrm{ X \;Total \;radioactivity\; counts \;applied}/\mathrm{adminstrated}}$$

The pharmacokinetic data of ^99m^Tc-ZT solution and ^99m^Tc-ZT-T6 gel was calculated from the biodistribution results as the maximum ^99m^Tc-ZT uptake % of the applied or the administrated dose per gram of blood or brain (%ID/ g) equivalent to C_max_ (%ID/ g) and the time to reach C_max_ is T_max_ (h). Non-compartmental analysis was adopted, via Kinetica^®^ software Ver. 5 (Thermo Fisher Scientific, Waltham, MA, USA), for the estimation of the area under the concentration–time curve from zero to 24 h (AUC_0–24 h_; %ID/ g) and the area under the curve from zero to infinity (AUC_0–∞_; %ID / g). AUC_0–∞_ values were utilized to calculate the blood and brain relative bioavailabilities of ^99m^Tc-ZT-T6 gel (test treatment), with respect to ^99m^Tc-ZT solution. The brain targeting efficiency (BTE%) of ^99m^Tc-ZT-T6 gel was calculated to test its capability in preferentially targeting the brain. BTE% was calculated as a ratio of ZT delivery to the brain after transdermal application (^99m^Tc-ZT-T6 gel) to its delivery following oral administration (^99m^Tc-ZT solution), according to the following equation:

5 $$\mathrm B\mathrm T\mathrm E\%=\frac{\frac{{\mathrm{AUC}0}-{\infty}\;brain}{{\mathrm{AUC}0}-{\infty}\;blood}\;transdermal}{\frac{{\mathrm{AUC}0}-{\infty}\;brain}{{\mathrm{AUC}0}-{\infty}\;blood}\;oral}\;\mathrm X\;100$$

## Results and discussion

### Development of ZT-loaded terpesomes

Terpesomes are ultra-deformable nanocarriers, capable of entrapping both hydrophilic and lipophilic drugs successfully with sufficient stability [22, 26]. Beside phospholipids, the main composition of terpesomes is terpenes and ethanol [12, 13]. Thin film hydration was adopted for the successful development of twenty-four ZT-loaded terpesomes using ZT, PC (at a drug: PC ratio of 1: 15 or 1: 7.5), terpene (fenchone, cineole, or limonene; 1% or 2%, w/v), SDC (0% or 0.1%, w/v) and ethanol (3%).

### In-vitro characterization of ZT-loaded terpesomes

The statistical significance of the four independent formulation variables on the characterization of the twenty-four developed terpesomes, was tested using Design-Expert^®^ software. Three numerical variables; drug: PC ratio (A), terpene concentration (C) and SDC concentration (D) and one categorical variable; terpene type (B) were investigated.

After statistical analysis, the equations of the five dependent responses (PS, ZP, EE%, DL% and Q_6h_) were developed, as following:

6 $$\mathrm{PS }= 290.10 - 23.31\mathrm{ A }+ 19.43\mathrm{ B}[1] + 4.22\mathrm{ B}[2] - 13.03\mathrm{ C }- 14.49\mathrm{ D}$$

7 $$\mathrm{ZP }= -46.05 + 1.59\mathrm{ A }- 0.15\mathrm{ B}[1] - 1.15\mathrm{ B}[2] - 1.18\mathrm{ C }- 3.27\mathrm{ D}$$

8 $$\mathrm{EE\% }= 67.75 - 6.05\mathrm{ A }+ 1.77\mathrm{ B}[1] + 1.89\mathrm{ B}[2] - 3.14\mathrm{ C}- 3.39\mathrm{ D}$$

9 $$\mathrm{DL\% }= 3.54+0.38\mathrm{ A }+ 0.098\mathrm{ B}[1] + 0.093\mathrm{ B}[2] - 0.62\mathrm{ C}- 0.22\mathrm{ D}$$

10 $$\mathrm{Q}6\mathrm{h }= 84.75 + 6.48\mathrm{ A }+ 0.83\mathrm{ B}[1] + 2.58\mathrm{ B}[2] + 2.39\mathrm{ C }+ 5.21\mathrm{ D}$$

where, A, B, C and D are coded variables, B[1], and B[2] are the coefficients of multi-level categorical variable.

### Particle size (PS), polydispersity index (PDI) and zeta potential (ZP)

**Fig. 1:**
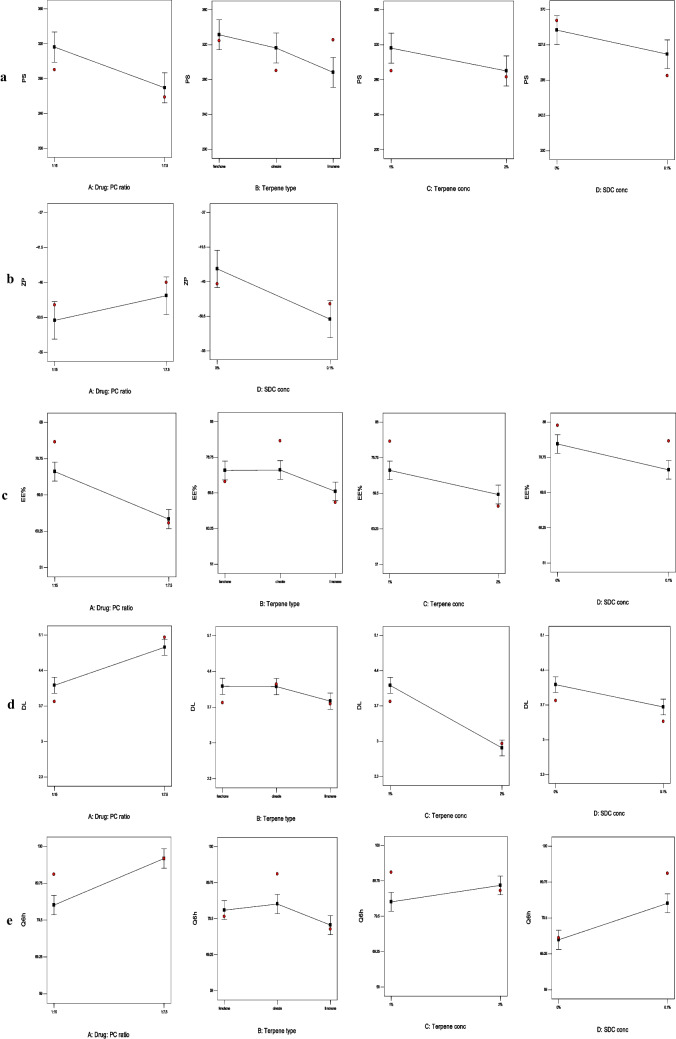
The influence of the significant formulation variables on the PS (**a**), ZP (**b**), EE% (**c**), DL % (**d**) and Q_6h_ (**e**) of ZT-loaded terpesomes

The PS of the developed ZT-loaded terpesomes varied from 208.2 ± 12.8 nm (T24) to 356.5 ± 6.7 nm (T5), Table 1. All the four variables; drug: PC ratio (*p* = 0.0001), terpene type (*p* = 0.005), (iii) terpene concentration (*p* = 0.01), and SDC concentration (*p* = 0.007) significantly influenced the PS, Fig. 1a.

**Table 1:** The Composition, and In-Vitro Characterization Data of the Developed ZT-loaded Terpesomes

**Systems**	**Composition**					**In-vitro characterization data**^**a**^				
	**Drug: PC ratio**	**Terpene type**	**Terpene concentration (%)**	**SDC concentration (%)**	**PS (nm)**		**ZP (mV)**	**EE (%)**	**DL (%)**	**Q6h (%)**
**T1**	1:15	fenchone	1	0	336.5 ± 13.2		-46.9 ± 3.4	79.5 ± 0.9	3.8 ± 0.1	75.4 ± 2.2
**T2**	1:15	fenchone	1	0.1	324.5 ± 9		-54.9 ± 2.8	72.4 ± 1.4	3.4 ± 0.2	80.1 ± 0.9
**T3**	1:15	fenchone	2	0	322.5 ± 7.5		-45.4 ± 1.2	76.7 ± 0.5	3.0 ± 0.1	79.1 ± 3
**T4**	1:15	fenchone	2	0.1	283.9 ± 13.8		-50.1 ± 5	69.1 ± 2	2.6 ± 0.2	84.7 ± 1.6
**T5**	1:15	cineole	1	0	356.5 ± 6.7		-46.3 ± 3.5	87.1 ± 1.1	4.1 ± 0.1	73.8 ± 2.8
**T6**	1:15	cineole	1	0.1	290.2 ± 10.1		-48.9 ± 2.9	83 ± 0.9	3.9 ± 0.2	92.2 ± 1.2
**T7**	1:15	cineole	2	0	330.4 ± 5		-42 ± 0.8	72.1 ± 1.8	2.8 ± 0.0	75.1 ± 0.7
**T8**	1:15	cineole	2	0.1	283.2 ± 9.4		-54.7 ± 1.5	66.1 ± 2.2	2.5 ± 0.1	86.9 ± 3.9
**T9**	1:15	limonene	1	0	348.2 ± 3.5		-39.7 ± 2	79 ± 2.7	3.8 ± 0.1	59.9 ± 0.5
**T10**	1:15	limonene	1	0.1	325.4 ± 6.4		-50 ± 3.9	67 ± 0.4	3.1 ± 0.0	76.5 ± 2.5
**T11**	1:15	limonene	2	0	266.1 ± 8.8		-43.9 ± 0.7	71.5 ± 3.1	2.8 ± 0.2	72.7 ± 1.3
**T12**	1:15	limonene	2	0.1	293.5 ± 10		-48.8 ± 1.4	62.3 ± 1.8	2.4 ± 0.1	82.8 ± 0.8
**T13**	1:7.5	fenchone	1	0	325 ± 6.3		-40.2 ± 2.1	68.2 ± 2.2	5.1 ± 0.2	84 ± 2
**T14**	1:7.5	fenchone	1	0.1	313.9 ± 9.5		-45.8 ± 0.8	64.9 ± 0.5	4.6 ± 0.1	92.6 ± 3.5
**T15**	1:7.5	fenchone	2	0	299 ± 14.2		-40.5 ± 3.7	65 ± 1.4	3.5 ± 0.1	88.7 ± 1.4
**T16**	1:7.5	fenchone	2	0.1	270.9 ± 7.5		-45.8 ± 1.3	60.5 ± 2.8	3.2 ± 0.0	100 ± 0.6
**T17**	1:7.5	cineole	1	0	300.3 ± 5.3		-41.4 ± 0.8	65.8 ± 1	4.9 ± 0.2	81 ± 2.7
**T18**	1:7.5	cineole	1	0.1	258.7 ± 7.4		-46 ± 2.6	62.4 ± 1.3	4.5 ± 0.1	96.6 ± 0.4
**T19**	1:7.5	cineole	2	0	294.8 ± 8		-47.1 ± 3.8	62.9 ± 0.7	3.4 ± 0.2	93.7 ± 1.4
**T20**	1:7.5	cineole	2	0.1	240.5 ± 11.3		-51.2 ± 2.2	57.9 ± 2.1	3.0 ± 0.0	99.3 ± 2.9
**T21**	1:7.5	limonene	1	0	243.9 ± 14.2		-37.4 ± 0.7	66.7 ± 0.9	4.9 ± 0.1	84.9 ± 1.5
**T22**	1:7.5	limonene	1	0.1	214.4 ± 7		-40.9 ± 1.4	54.9 ± 1.6	3.9 ± 0.1	91.3 ± 3.7
**T23**	1:7.5	limonene	2	0	231.9 ± 9.5		-42.5 ± 3	59.4 ± 2.7	3.2 ± 0.1	86.1 ± 2.1
**T24**	1:7.5	limonene	2	0.1	208.2 ± 12.8		-54.7 ± 4.2	51.9 ± 0.5	2.7 ± 0.0	96.5 ± 0.7

^a^Values are expressed as mean ± S.D. (n = 3)

*PS* particle size, *ZP* zeta potential, *EE%* drug entrapment efficiency percentage, *Q*_*6h*_ drug released % after 6 h

Direct relation was observed between PS and the lipid content. Terpesomes prepared at a drug: lipid ratio of 1: 15, showed significantly greater sizes than their corresponding terpesomes prepared at lower lipid content. Greater mass transfer resistances, due to higher viscosities at higher lipid content could be the reason behind the larger vesicles [30, 36]. Similar findings were previously reported upon increasing the PC concentration [22, 26].

Concerning the terpene type, the PS of the developed terpesomes was inversely correlated to the terpenes’ lipophilicity. By applying LSD test, fenchone-based vesicles had significantly greater PS compared to cineole- and limonene-based ones, (log P = 2.13, 2.82, and 4.83, respectively). This could be due to the repulsion between the lipophilic nature of terpesomes and the hydrophilic nature of fenchone [22]. Same finding was recorded by Albash et al. in 2021 [26]. Also, Saffari et al. reported that invasomes prepared with limonene had smaller sizes than others prepared with cineole [37].

Significant decrease in PS was observed upon increasing the concentration of terpene from 1% w/v to 2% w/v. This is well in line with the results of Albash et al. who assumed that the reason was Ostwald ripening inhibition, as a result of the reduced interfacial tension as well as viscosity of the internal phase to the external dispersion medium [38].

Statistical results declared the influence of SDC concentration on PS, where upon the addition of 0.1% w/w SDC, smaller vesicles were formed due to reduced surface tension [39] and micelles formation. This result runs with that published by Albash et al. who revealed smaller olmesartan medoxomil loaded transethosomes [40], as well as smaller fenticonazole nitrate loaded terpene-based liposomes [23] upon increasing the SDC amount.

PDI results were directly correlated with the PS of the developed terpesomes. PDI values ranged from 0.12 ± 0.04 (T24) to 0.5 ± 0.10 (T1), data not presented. Low PDI values indicate homogenous PS distribution.

Zeta potential reveals the physical stability by estimating the repulsion between vesicles in the dispersions, where high absolute ZP values (≥ 30 mV) indicate stable dispersions with no particle aggregation [30, 41]. The developed ZT-loaded terpesomes showed negative ZP values, ranging from -37.4 ± 0.7 mV (T21) to -54.9 ± 2.8 mV (T2); Table 1. ANOVA findings showed that drug: PC ratio (*p* = 0.02), and SDC concentration (*p* = 0.0001) had significant impact on the ZP of the developed terpesomes, Fig. 1b.

The absolute ZP values are positively correlated to PC, SDC and ethanol. Incorporating PC exhibits negative charge, via orienting the phosphatidyl group (negatively charged) to the outside, while the choline group (positively charged) to the inside [22, 26]. Previous studies reported significant elevation of absolute ZP values of the prepared bilosomes [7, 42], and terpesomes [23], upon increasing the concentration of SDC due to its anionic nature. Also ethanol plays a great role in the stabilization of the vesicular dispersions, via imparting negative charge due to its anionic nature [22, 23].

### ZT Entrapment efficiency (EE%) and drug loading (DL%)

The EE% of ZT in the developed terpesomes varied from 51.9 ± 0.5% (T24) to 87.1 ± 1.1% (T5) and DL% of ZT in the developed terpesomes varied from 2.4 ± 0.1% (T12) to 5.1 ± 0.2% (T13); Table 1. In our study, the four independent variables; drug: PC ratio (*p* < 0.0001, *p* < 0.0001), terpene type (*p* = 0.004, *p* = 0.0206), terpene concentration (*p* = 0.0002, *p* < 0.0001) and SDC concentration (*p* < 0.0001, *p* < 0.0001), significantly affected the ZT EE% and DL%, respectively, Fig. 1c, d.

Significantly increase in ZT EE% was revealed upon increasing the PC concentration (switching drug: PC ratio from 1: 7.5 to 1:15). Enhanced entrapment and minimized drug leakage might be related to the surfactant properties of PC, thus forming coherent layers around the terpesomes [22, 26]. Furthermore, increasing the PC concentration increases the viscosity of the dispersion, which minimizes the diffusion of drug externally [22, 30]. It is worth mentioning that ethanol has a great role in enhancing ZT EE%, via increasing the drug solubility. On the other hand, switching drug: PC ratio from 1: 7.5 to 1:15 led to decrease in ZT DL%, despite increase in ZT EE%. This finding might to correlated to the insufficient increase in drug EE% upon duplicating the lipid amount (shifting the lipid amount from 150 to 300 mg).

ANOVA results declared that the terpene type significantly affected ZT EE% and DL%. By applying LSD test, fenchone-based terpesomes showed maximum EE% and DL% compared to cineole-, and limonene-based vesicles, respectively. This result was explained in the light of the different lipophilicities of the used terpenes; fenchone (most hydrophilic among the used terpenes; log *P* = 2.13), cineole (log *P* = 2.82), and limonene (most lipophilic among the used terpenes; log *P* = 4.83) [22, 24]. Inverse correlation was observed between the lipophilicity of the used terpene and the ZT solubilization and EE% and DL%. ZT, being hydrophilic, showed highest EE% and DL% when incorporated with fenchone-based terpesomes. The obtained results are in harmony with that detected by Vidya et al. who demonstrated that maximum EE% of anastrozole (hydrophilic drug) was achieved upon using fenchone rather than other lipophilic terpenes [43]. This is also comparable to that achieved by Albash et al. who reported highest moxifloxacin hydrochloride (hydrophilic drug) EE% upon incorporation in fenchone-based terpesomes [26]. On the contrary, highest EE% of lipophilic drugs as dapsone [24], finasteride [44], and curcumin [25] was reported with limonene-based terpesomes.

Concerning terpene concentration, significant decrease in ZT EE% and DL% were found upon shifting the terpene concentration from 1% w/v to 2% w/v. Dragicevic-Curic et al. believed that the higher the terpene concentration, the higher the fluidity of the terpesomes, the lower the EE% and DL% of drugs [45]. Moreover, decreased EE% could be related to pore formations and lipid bilayer destabilization of the developed terpesomes, upon increasing the terpene concentration [38].

SDC negatively influenced the ZT EE% and DL%, where lower ZT EE% and DL% were revealed upon the addition of SDC, owing to its effect on decreasing the PS. The result in the present study is consistent with the previous observation of Albash et al. who reported pore formation in the terpesomal bilayers, and consequently lower fenticonazole nitrate entrapment on increasing SDC amount [23]. Elsharkawy et al. also reported that SDC had negative significant impact on the DL% of clozapine in lecithin-based mixed polymeric micelles [29].

### In-vitro ZT release studies

**Fig. 2:**
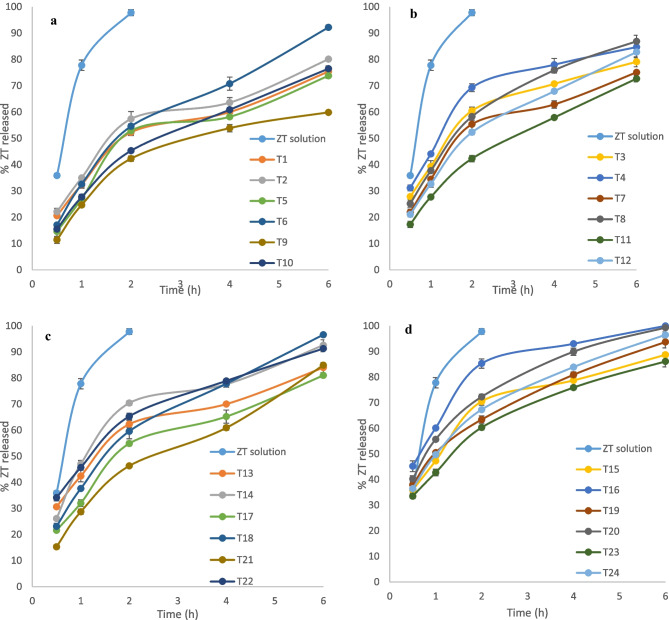
In-vitro drug release profiles from ZT-loaded terpesomes prepared at a drug: PC ratio of 1: 15 & terpene concentration of 1% (**a**), at a drug: PC ratio of 1: 15 & terpene concentration of 2% (**b**), at a drug: PC ratio of 1: 7.5 and terpene concentration of 1% (**c**), and at a drug: PC ratio of 1: 7.5 and terpene concentration of 2% (**d**), in comparison to ZT aqueous solution, in phosphate buffer saline (pH 7.4) at 37 ± 0.5 °C (mean ± S.D., n = 3)

The ZT release profiles of the developed ZT-loaded terpesomes prepared at ZT: PC ratio of 1: 15 (T1-T12) and at ZT: PC ratio of 1: 7.5 (T13-T24), compared to ZT aqueous solution are shown in Fig. 2. Almost complete ZT release, from its aqueous solution, was reached in 2 h; confirming the absence of any hinderance caused by the cellulose dialysis membrane. On the contrary, biphasic ZT release profiles were revealed with ZT-loaded terpesomes. An initial burst ZT release phase occurred within the first 0.5–1 h, followed by a slower prolonged ZT release phase. Firstly, the initial phase could be correlated to the surface/ adsorbed ZT, taking in consideration the ZT’s hydrophilic nature, while the slower prolonged phase might be contributed to the partitioning of the ZT entrapped within the terpesomes into the external release media [30]. These biphasic profiles are beneficious for the anti-migraine drugs, where the initial burst phase might provide rapid onset of action and the slower prolonged phase could maintain the pain relief effect over 6 h [7].

ZT released percentages after 6 h (Q_6h_), chosen as discriminators of ZT release profiles, were tabulated; Table 1. ANOVA results confirmed the significant impacts of ZT: PC ratio (*p* < 0.0001), terpene type (*p* = 0.01), terpene concentration (*p* = 0.005), and SDC concentration on Q_6h_ (*p* < 0.0001), Fig. 1e.

Terpesomes prepared at a ZT: PC ratio of 1: 15 showed significantly lower Q_6h_ than their corresponding values of the terpesomes prepared at a ZT: PC ratio of 1: 7.5. Slower ZT diffusion rates were mainly attributed to the lipophilic matrix, where lipid coating restrains the diffusion and release of drugs. Such finding coincides with that reported about lipid-polymer hybrid particles loaded with vardenafil hydrochloride [30]. Also, our results were supported by Tawfik et al., who stated that the drug release from agomelatine-loaded invasomal systems developed at a drug: PC ratio of 1:10 were significantly slower than their corresponding systems developed at a drug: PC ratio of 1:7.5 [22].

Fenchone-based terpesomes revealed significantly higher Q_6h_. This is mainly referred to the EE% results, where the lipophilicity of the used terpene is inversely related to the ZT EE% as well as the ZT release. By applying LSD test, fenchone-based terpesomes showed maximum EE% and fastest drug release, compared to cineole- and limonene- based ones, respectively. ZT concentration gradient between the developed terpesomes and the release medium was considered the driving force for drug release [22].

The terpene concentration revealed significant positive contribution on Q_6h_. Faster ZT release into the release medium was found upon increasing the terpene concentration from 1% w/v to 2% w/v. This is well in line with the results obtained from invasomal systems developed at terpene concentrations of 0.75% and 1.5% w/v [22]. Current results could to referred to the influence of particle size on the rate of drug release, where the higher the terpene concentration, the smaller the particle size, the greater the surface area exposed to the release medium, and hence the faster the release rate [30].

ANOVA findings declared that SDC concentration positively influenced Q_6h_. Significantly faster ZT drug release rates were observed upon the addition of SDC. Owing to the effect of SDC on decreasing the surface tension, and hence decreasing the PS, SDC-based terpesomes exhibited faster release rates.

### Selection of the optimum ZT-loaded terpesomes

T6; prepared employing 1: 15 as drug: PC ratio, cineole terpene at 1% w/v concentration and SDC concentration of 0.1% w/w, achieved the highest desirability value of 0.85. Thus, further investigations were adopted for T6.

### Characterization of the optimum ZT-loaded terpesomes

#### Transmission electron microscopy (TEM)

**Fig. 3:**
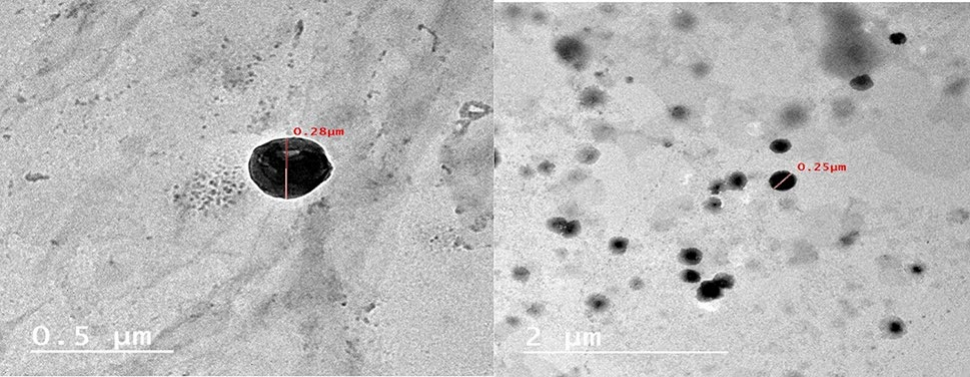
Transmission electron micrograph of ZT-loaded terpesomes (T6)

The morphological assessment of the optimum ZT-loaded terpesomes (T6) via TEM, showed spherically shaped non-aggregating nano-vesicles (Fig. 3), whose diameters were in good agreement with photon correlation spectroscopy observations (Table 1).

#### Differential scanning calorimetry (DSC) studies

**Fig. 4:**
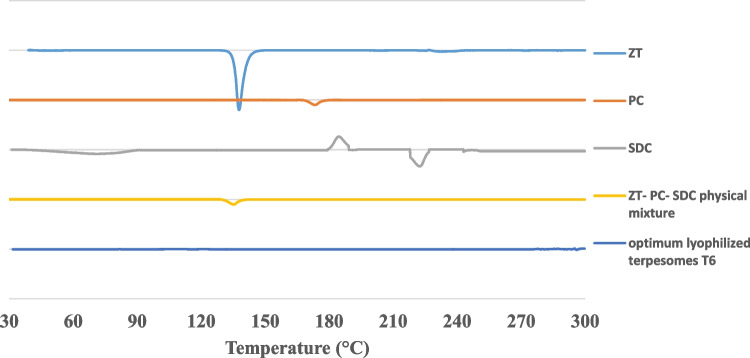
DSC thermograms

Figure 4 illustrated the DSC thermograms of pure ZT, PC, SDC, ZT-PC-SDC physical mixture and T6 lyophilized terpesomes. The crystallinity of ZT was evidenced by its specific sharp melting endothermic peak at 137.8 °C [7, 20]. ZT’s melting peak disappeared in the thermogram of T6, indicating amorphization and successful entrapment of ZT inside the terpesomal vesicles [46].

### In-vivo studies

#### In-vivo histopathological studies

**Fig. 5:**
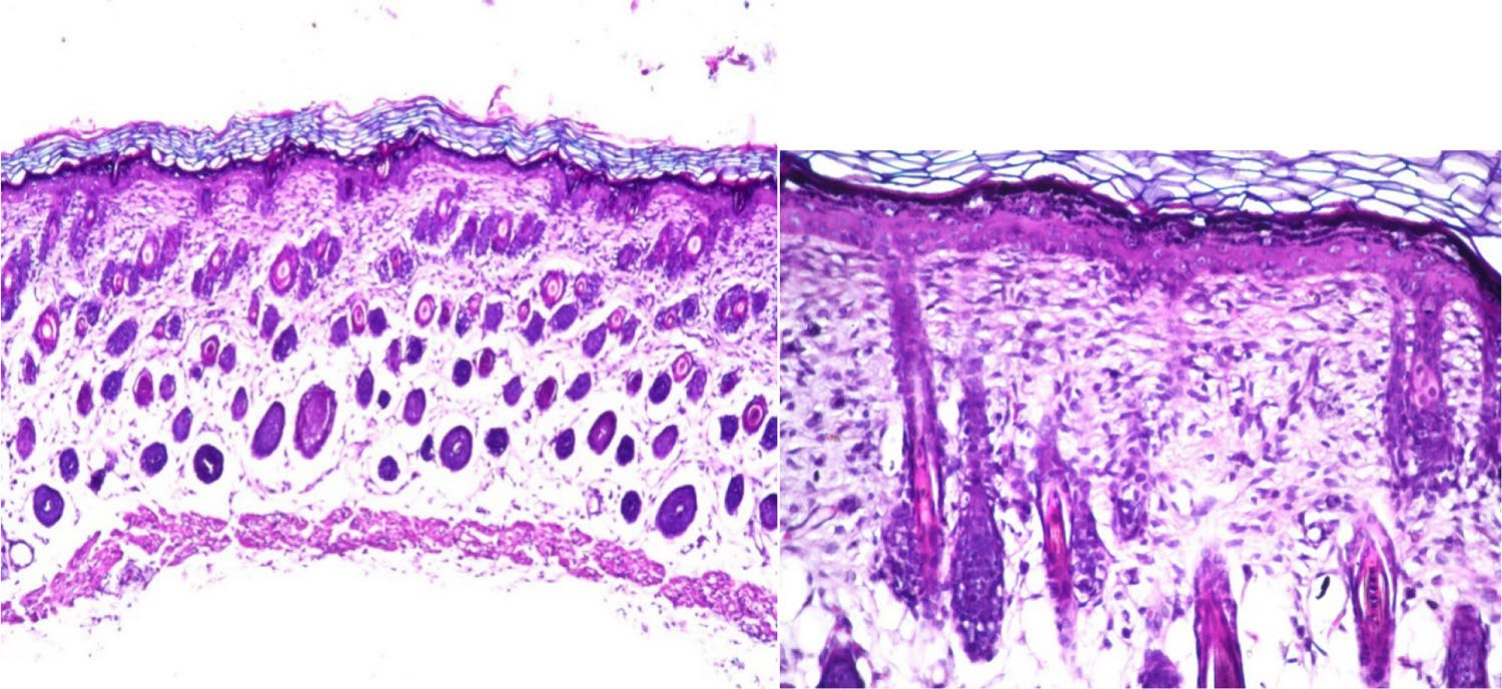
Histopathological photomicrographs (hematoxylin and eosin stained) of rat skin treated with the optimum terpesomes (T6), with magnification power of × 16 and × 40, respectively

Figure 5 illustrated the histopathological photomicrographs of hematoxylin and eosin-stained rat skin treated with T6, which showed normal structure including all the skin layers (epidermis, along with underlying dermis, followed by adipose subcutis and muscular layer), free from any histopathological alteration, irritation, nor skin inflammation. These findings confirmed that ZT terpesomes revealed acceptable safety when transdermally applied.

### In-vivo biodistribution studies

#### Radio-labeling

**Fig. 6:**
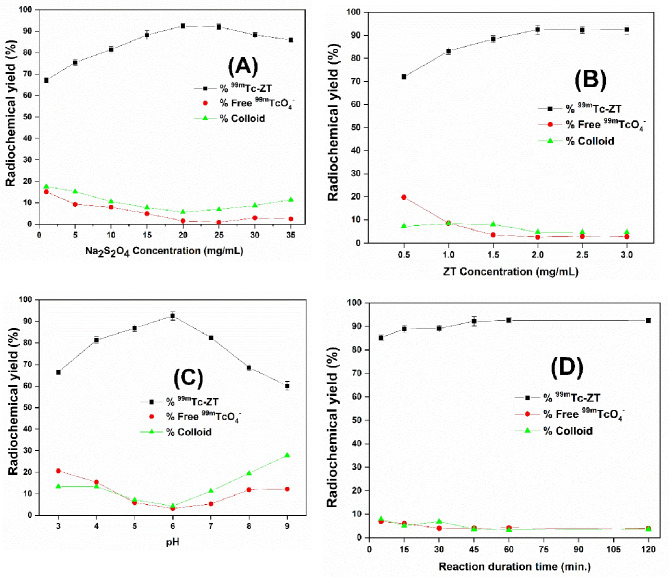
Fluctuation of radiochemical yield of ^99m^Tc-ZT dispersion as a function of various reaction factors; sodium dithionite concentration (**A**), ZT concentration (**B**), pH (**C**), and reaction duration time (**D**)

**Fig. 7:**
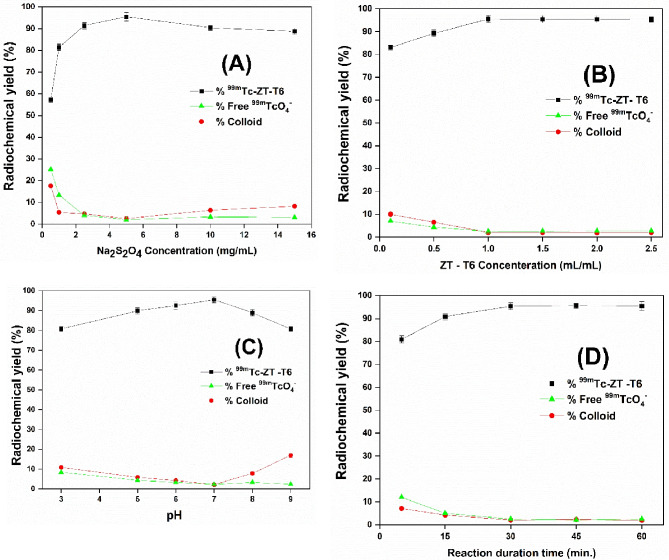
Fluctuation of radiochemical yield of ^99m^Tc-ZT-T6 as a function of various reaction factors; sodium dithionite concentration (**A**), ZT-T6 concentration (**B**), pH (**C**), and reaction duration time (**D**)

ZT was successfully radiolabeled with a high radiochemical yield (RCY) value of 92.5 ± 1.2%, employing 20 mg sodium dithionite with 2 mg ZT, at pH 6 and 25 °C for 45 min. The in-vitro stability of the developed ^99m^Tc-ZT complex was tested and showed sufficient stability for up to 24 h. ^99m^Tc-ZT-T6 complex was efficiently synthesized in excellent yield (95.5 ± 1.5%), under optimized conditions via incubating the selected terpesomes (1 mL) with Na_2_S_2_O_4_ (5 mg), and freshly eluted technetium Tc-99 m pertechnetate (^99m^TcO_4_^−^, 100 MBq; 0.1 mL) at 25 °C for 30 min and the pH was adjusted to 7. The fluctuations of radiochemical yield (RCY) % of ^99m^Tc-ZT solution and ^99m^Tc-ZT-T6 were adopted, as function of various reaction factors; sodium dithionite concentration, ZT concentration, reaction medium pH, and reaction duration time (Figs. 6 and 7).

### In-vivo biodistribution of oral ^99m^Tc-ZT solution and transdermal ^99m^Tc-ZT-T6 terpesomal gel

**Fig. 8:**
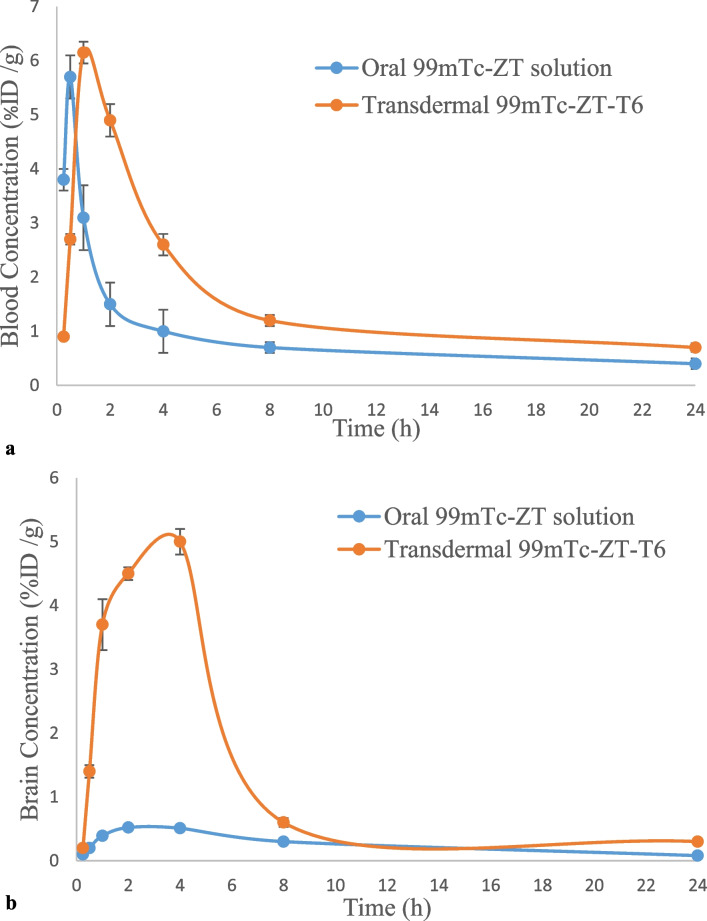

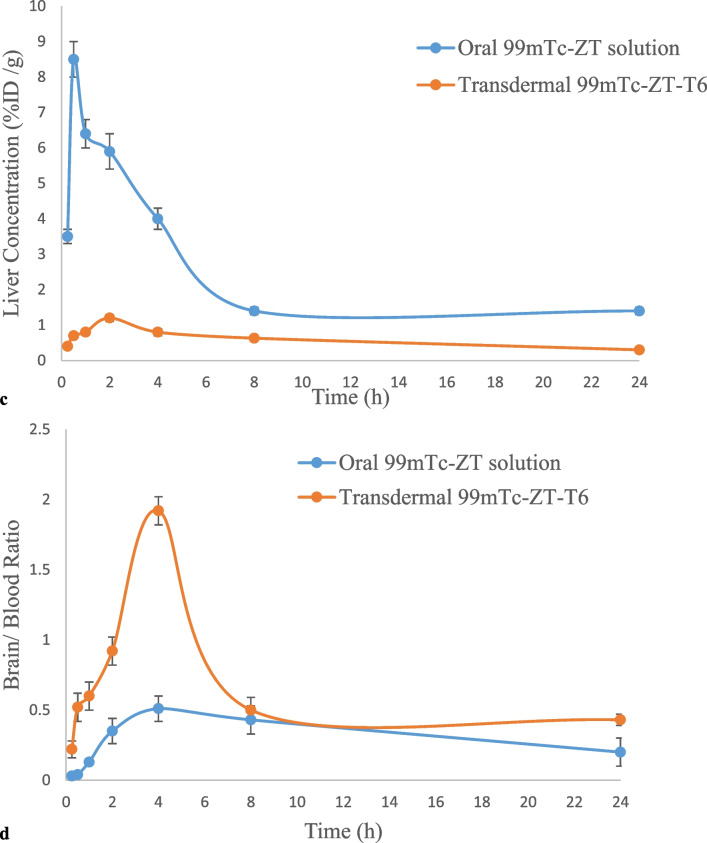
**a** ^99m^Tc-ZT concentrations in blood at different time intervals following the oral administration of ^99m^Tc-ZT dispersion and the transdermal application of ^99m^Tc-ZT-T6 gel in mice (mean ± SD, n = 3). **b** ^99m^Tc-ZT concentrations in brain at different time intervals following the oral administration of ^99m^Tc-ZT dispersion and the transdermal application of ^99m^Tc-ZT-T6 gel in mice (mean ± SD, n = 3). **c** ^99m^Tc-ZT concentrations in liver at different time intervals following the oral administration of ^99m^Tc-ZT dispersion and the transdermal application of ^99m^Tc-ZT-T6 gel in mice (mean ± SD, n = 3). **d** Brain/ blood ratios at different time intervals following the oral administration of ^99m^Tc-ZT dispersion and the transdermal application of ^99m^Tc-ZT-T6 gel in mice (mean ± SD, n = 3)

The in-vivo stability of the developed ^99m^Tc-ZT complex was confirmed by its low stomach uptake after oral administration of ^99m^Tc-ZT solution and transdermal application of ^99m^Tc-ZT-T6 gel [18, 47]. The ^99m^Tc-ZT uptakes in brain, blood and liver were estimated, at predesigned time intervals up to 24 h, that were equivalent to the ZT concentrations in organs or fluid. Following the oral administration of ^99m^Tc-ZT solution and the transdermal application of ^99m^Tc-ZT-T6 gel, ZT concentrations (%ID /g; mean ± SD, n = 3) in blood, brain, and liver were assessed and illustrated in Fig. 8a, b, c, respectively. Pharmacokinetic parameters (C_max_, T_max_, AUC_0–24 h_ and AUC_0-∞_) of both treatments were calculated by Kinetica^®^ software Ver. 5 and displayed in Table 2.

**Table 2:** The Pharmacokinetic Parameters and the Relative Bioavailability Percentages of ZT Following the Transdermal Application of ^99m^Tc-ZT-T6 Gel System and the Oral Administration of ^99m^Tc-ZT solution in Mice (Mean ± S.D., n = 3)

Pharmacokinetic parameters	Transdermal		Oral	
	**Brain**	**Blood**	**Brain**	**Blood**
**C**_**max**_** (%ID /g)**	5 ± 0.10	6.15 ± 0.18	0.52 ± 0.01	5.70 ± 0.09
*T_**max**_** (h)**	4 (2–4)	1 (0.5–1)	2 (1–2)	0.5 (0.5–0.5)
**AUC**_**0-24 h**_** (%ID.h /g)**	29.75 ± 3.40	38.60 ± 2.90	6.34 ± 1.90	20.41 ± 2.20
**AUC**_**0-∞**_** (%ID.h /g)**	38.32 ± 4.80	49.62 ± 4.10	7.25 ± 2.30	29.58 ± 3
**Relative Bioavailability (%)**	528.55	167.75	-	

*ZT* zolmitriptan, *C*_*max*_ peak plasma concentration, *T*_*max*_ time to reach C_max_, *AUC*_*(0–24 h)*_ area under the curve from zero to the last sampling point, *AUC*_*(0–∞)*_ area under the curve from zero to infinity

^*^Median (range)

ZT was rapidly absorbed and reached maximum ^99m^Tc-ZT blood concentration (C_max_) of 5.70 ± 0.09%ID/ g at 0.5 h post oral administration, after which ZT concentration declined fast in the following hours. However, significantly (*p* < 0.05) higher blood C_max_ was achieved (6.15 ± 0.18%ID/ g) at higher T_max_ (1 h) post the transdermal application of ^99m^Tc-ZT-T6 gel, after which slower decrease in drug concentration was revealed; Fig. 8a. These findings were reflected on the results concerning brain samples; where significantly (*p* < 0.05) higher maximum brain concentrations wa reached following the transdermal application of ^99m^Tc-ZT-T6 gel (5 ± 0.1%ID/ g) at T_max_ (4 h), compared to the oral administration of ^99m^Tc-ZT solution (0.52 ± 0.01%ID/ g) at T_max_ (2 h); Fig. 8b. Significant higher T_max_ values of transdermal ^99m^Tc-ZT-T6 gel could be due to the fact that the administration skin surface acts as a reservoir for ^99m^Tc-ZT-T6, maintaining effective drug concentrations for sufficient time [48]. Compared to the oral administration of ^99m^Tc-ZT solution, the transdermal application of ^99m^Tc-ZT-T6 showed significantly (*p* < 0.05) lower liver concentrations, which evidenced bypassing the extensive first pass metabolism in the liver, (Fig. 8c). Moreover, brain/blood ratios were calculated. The potentiality of ^99m^Tc-ZT-T6 for brain targeting was confirmed by significantly higher brain/ blood ratios at all-time points post transdermal application, in contrast to oral administration; Fig. 8d.

Maximum ZT brain concentration (5 ± 0.1%ID/ g) and highest brain/blood ratio (1.92 ± 0.1) were attained at 4 h post transdermal application of ZT-loaded terpesomes (T6), with lower hepatic exposure. Dragicevic-Curic et al. emphasized the enhanced permeation of terpesomes through the stratum corneum, either being intact or fragmented along permeation [49]. Owing to their elasticity and the osmotic gradient, small intact terpesomes reveal prolonged and deep permeation through the stratum corneum layers. Fragmented terpesomes alter the lipid lamellae via mixing of phospholipids and terpenes with the stratum corneum lipids. Furthermore, ethanol also plays a great role in enhancing drug permeation via disarranging the stratum corneum’s bilayer structure. The obtained results were in harmony with that reported for agomelatine after the transdermal application of invasomal gel systems [22].

Transdermal ^99m^Tc-ZT-T6 significantly (*P* < 0.01) improved the ZT bioavailability, compared to oral ^99m^Tc-ZT solution. Significant improvement of ZT blood and brain relative bioavailabilities were achieved; 1.68-folds and 5.29-folds (based on the estimated AUC_0-∞_ values), respectively (Table 2). High brain targeting efficiency (BTE% = 315%) of ^99m^Tc-ZT-T6 was revealed, which confirmed its successful capability in crossing the blood brain barrier (BBB), as well as higher distribution in brain compared to ^99m^Tc-ZT solution. This improvement could be correlated to various factors; (i) the lipid nature of the vesicular system enhancing its affinity in crossing the skin as well BBB either intact or fragmented [20, 49] (ii) the augmented effects of terpene, ethanol and SDC in enhancing skin permeation [22], (iii) the small particle size, large surface area and elasticity allowing the intimate contact and penetration through the skin layers [23], and (iv) bypassing the hepatic first pass metabolism [46, 50].

The transdermal route is characterized by numerous points of strength that can enhance ZT bioavailability; i) it conveys drug directly into the systemic circulation avoiding the portal circulation and consequently first pass metabolism, ii) it avoids the GIT which diminishes the negative effect of the migraine associated GIT disturbance on ZT bioavailability, and iii) it provides consistent drug levels in blood via controlled delivery, that decreases the dosing frequency as well as associated side effects [10]. Stratum cornuem (SC), the upper layer of skin, represents the main barrier for the transdermal absorption of drugs [11]. Therefore, utilizing penetration enhancer that facilitates drug permeation through the SC is considered crucial to accomplish successful transdermal absorption.

Previous studies explored the transdermal route of administration for enhancing the bioavailability of ZT via nanostrucutured lipid carriers [20], niosomal emulgel [8], adhesive patch [21]. However, only plasma concentrations of ZT were determined rather than concentrations of ZT in the target organ (brain) [8, 20, 21]. Also, Mohamed et al. [8] and Liu & Fang [21] did not conduct histopathological studies for safety assessment after the transdermal application. Also, it is worth mentioning that reaching C_max_ at 8 h after the application of niosomal emulgel for an anti-migraine drug is not advantageous [8]. The current study did not assess the drug concentration in blood only, but also in brain and liver. Significant enhancement of ZT’s bioavailability was evidenced by rapid achievement of C_max_ in blood (1 h post application), significant low ZT concentrations in liver and high brain relative bioavailability (5.29-folds) as well as brain targeting efficiency (315%). On the other hand, in-vivo histopathological studies were conducted to confirm the safety of the transdermally applied terpesomal gel.

## Conclusion

ZT terpesomes were developed successfully by adopting thin film hydration technique, via a 2^3^.3^1^ full factorial design. T6 system, comprising 1: 15 as drug: PC ratio, cineole terpene at 1% w/v concentration and SDC concentration of 0.1% w/w, was the optimum system with respect to spherical PS (290.2 nm), ZP (-48.9 mV), PDI (0.3), EE% (83%), DL% (3.9%) and Q_6h_ (92.2%), crystallinity and morphology. The in-vivo histopathological studies evidenced the safety of the developed T6 terpesomes. Compared to oral ^99m^Tc-ZT solution, ^99m^Tc-ZT-T6 gel showed maximum brain concentration (5 ± 0.1%ID/ g) with highest brain to blood ratio of 1.92 ± 0.1 at 4 h post transdermal application. The in-vivo biodistribution studies in mice evidenced the potentiality of T6 system in improving ZT relative bioavailability (529%) and targeting the brain efficiently (315%) for sufficient time. For confirming our results, clinical studies need to be conducted.

## Data Availability

All data generated or analyzed during this study are included in this published article.
